# Label-Free Detection of T4 Polynucleotide Kinase Activity and Inhibition via Malachite Green Aptamer Generated from Ligation-Triggered Transcription

**DOI:** 10.3390/bios13040449

**Published:** 2023-03-31

**Authors:** Jingyi Si, Wei Zhou, Ying Fang, Da Zhou, Yifan Gao, Qunyan Yao, Xizhong Shen, Changfeng Zhu

**Affiliations:** 1 Department of Gastroenterology and Hepatology, Zhongshan Hospital, Fudan University, Shanghai 200032, China; 2 Department of School and Nutrition, Shanghai Yangpu District Center for Disease Control and Prevention, Shanghai 200090, China

**Keywords:** T4 polynucleotide kinase, malachite green aptamer, T7 transcription

## Abstract

Polynucleotide kinase (PNK) is a key enzyme that is necessary for ligation-based DNA repair. The activity assay and inhibitor screening for PNK may contribute to the prediction and improvement of tumor treatment sensitivity, respectively. Herein, we developed a simple, low-background, and label-free method for both T4 PNK activity detection and inhibitor screening by combining a designed ligation-triggered T7 transcriptional amplification system and a crafty light-up malachite green aptamer. Moreover, this method successfully detected PNK activity in the complex biological matrix with satisfactory outcomes, indicating its great potential in clinical practice.

## 1. Introduction

Despite constant efforts to develop novel therapeutic strategies, cancer remains the second leading cause of death globally. One of the most commonly used anti-tumor approaches is to damage the genomic DNA of tumor cells, thereby causing their death. A considerable number of current first-line anti-tumor regimens act by damaging the genomic DNA of tumor cells via radiation or chemical drugs to inhibit their proliferation [1,2]. For example, γ ray, used in radiotherapy and chemotherapeutic agents such as Nitrosourea, can induce strand breaks accompanied by the generation of hydroxyl at the 5′-end of DNA, which damages the genetic material of tumor cells. The phosphorylation-dependent rehabilitation of DNA damage refers to a complex biological process that involves the phosphorylation of DNA through various kinases at the site of DNA damage and the consequent recruitment of repair proteins, ultimately resulting in the repair of damaged DNA. This process plays a crucial role in the repair of damaged DNA in cells. However, rehabilitation can also lead to the attenuation of the cytotoxicity of chemo- and radio-therapeutic strategies, resulting in treatment resistance and tumor relapse [3,4,5].

The phosphorylation of DNA, completed by multifarious kinases, can occur at different sites. Among them, the phosphorylation of the DNA 5’ terminal hydroxyl group is one of the most critical phosphorylation processes in life activities. The disturbance phosphorylation process of the 5′-hydroxyl termini of DNA can cause the destruction of genetic materials, which may lead to the occurrence of diseases such as Werner Syndrome and ataxia-oculomotor apraxia type 4 [6,7]. However, the phosphorylation of the 5′-hydroxyl termini of damaged DNAs and its consequent rehabilitation is a significant obstacle in successful tumor treatment since it contributes to the resistance of anti-tumor therapies. Polynucleotide kinase (PNK), which catalyzes the transfer of the γ-phosphate residue of ATP to the 5ʹ-hydroxyl group of damaged DNAs and is necessary for ligation-based DNA repair, has been found to be a key enzyme in this process since 1965 [8,9]. Therefore, understanding the role of PNK in the phosphorylation-dependent rehabilitation of DNA damage is essential for the development of effective anti-tumor therapies. The activity assay and inhibitor screening for PNK can contribute to the prediction and improvement of tumor treatment sensitivity, respectively.

Conventional methods for PNK activity detection, including polyacrylamide gel electrophoresis (PAGE) [10] and autoradiography [11], suffer the intrinsic drawbacks of high cost, radioactive contamination, and the requirement of skillful technicians. Recently, diverse colorimetric [12], electrochemical [13,14,15], and fluorescent methods [16,17] have been developed to overcome the above shortcomings. Among them, homogeneous fluorescence assays are widely considered due to their advantages of simplicity and efficiency. However, without a signal amplification process, most fluorescence-based assays are limited in their sensitivity [18]. To improve the sensitivity of PNK activity analysis, signal amplification techniques of nucleic acids, such as rolling circle amplification (RCA) [19,20] and nickase-assisted signal amplification, have been employed [21,22]. Signal amplification strategies making use of DNA polymerase require repeated primers; however, too much of a high concentration of the primer affects the specificity of amplification reactions [23]. Nickase-assisted signal amplification always relies on the dual enzyme system and the assistance of costly fluorescent-labeled probes, which are always limited by the demand for precision synthesis instruments and easily suffer from fluorescence photobleaching. Therefore, the development of practical and accurate signal amplification methods for the detection of PNK activity still remains a great challenge.

T7 RNA polymerase is a frequently used enzyme in the field of nucleic acids research due to its robust and specific activity alongside Taq DNA polymerase. T7 RNA polymerase can efficiently and specifically catalyze in vitro RNA synthesis from DNA templates downstream to the T7 promoters. One of the key advantages of T7 RNA polymerase over DNA polymerase is its ability to continuously and isothermally generate separate product RNA chains from a single DNA template [24]. Additionally, with recent advances in the development of light-up RNA aptamers [25,26], T7 RNA polymerase catalyzing RNA synthesis has been recruited as a valuable signal amplification system for biomolecule detection [27,28,29]. One such RNA aptamer is the malachite green aptamer (MGA), which is an in vitro selected RNA sequence with the ability to specifically form a complex with malachite green (MG) and consequently induce more than 2000-fold fluorescence compared with the neglectable fluorescence of a free MG molecule [30,31]. In this work, the MGA-producing-T7 in vitro transcription system could be generated via ATP-dependent ligation, which is triggered by PNK-catalyzed phosphorylation. Then, the T7 transcription system can constantly produce lots of MGA sequences and amplify the fluorescence signal. Combining the robust T7 transcriptional amplification with the crafty light-up MGA system, we herein developed a simple, extremely low-background, and label-free method for PNK activity detection as well as PNK inhibitor screening. Moreover, this method successfully detected PNK activity in the complex biological matrix with satisfactory outcomes, proving its promising application in the biomedical field.

## 2. Materials and Methods

### 2.1. Apparatus

The fluorescence emission spectra were recorded by a Lengguang F97Pro fluorophotometer (shanghai Lengguang Technology Co., Ltd., Shanghai, China) with an excitation wavelength of 616 nm. All the enzymatic reactions were conducted at the optimal temperature in a TC1000-G PCR Thermal Cycler (DLAB Scientific Co., Ltd., Beijing, China). The cell lysate was obtained using an FS600N ultrasonic processer (SXSONIC, Shanghai, China).

### 2.2. Materials and Reagents

T4 polynucleotide kinase (T4 PNK), T4 DNA ligase, T7 RNA polymerase, bovine serum albumin (BSA), Bst 2.0 WarmStart DNA polymerase, terminal transferase (TdT), Nt. AlwI, adenosine 5’-triphosphate (ATP), and ribonucleotide (rNTP) solution mix were obtained from New England Biolabs (MA, USA). The C-reactive protein (CRP) was purchased from Cusabio (Wuhan, China). Fetal bovine serum (FBS), high glucose Dulbecco’s modified Eagle’s medium (DMEM), penicillin/streptomycin solution, and phosphate-buffered saline (PBS) buffer were purchased from Gibco (Waltham, MA, USA). All the oligonucleotides (the sequences of these oligos are listed in Table 1) and other chemicals were obtained from Sangon Biotechnology Co., Ltd. (Shanghai, China) and used without further purification. All solutions were prepared using deionized water taken from an EVOQUA Ultra Clear water purification system (Barsbüttel, Germany) with a resistivity of 18.2 MΩ·cm^−1^. The tips and tubes used in this work were RNase-free.

### 2.3. Amplified Fluorescence Assay for T4 PNK-Catalyzed Phosphorylation

In a typical experiment, the whole detection process is divided into four parts: the phosphorylation process, the ligation process, the transcription process, and the fluorescence measurement process. In the phosphorylation process, different concentrations of T4 PNK are incubated with 1 mM promoter probes and 1 mM ATP for 2 h at 37 °C in a 20 μL of 1× T4 PNK buffer, followed by inactivation through heating to 65 °C for 20 min. In addition, the 5′ phosphorylated promoter probe was used as a positive control in feasibility verification and inhibitor screening experiments. The reaction system for the ligation process consists of the phosphorylation products and 200 nM of the other two probes with 10 units of T4 DNA ligase in a 1× T4 DNA ligase reaction buffer. Subsequently, the T4 DNA ligase catalyzes the reaction of the three probes at 16 °C for 1 h, which is then terminated by heating to 65 °C for 10 min. After the ligation process, 100 units of T7 RNA polymerase, 20 units of RNase inhibitor, 0.8 of μmol rNTP, and 5 μL of a 10× RNA pol reaction buffer were added into the mixture taking the volume up to 100 μL with DEPC-treated water. The transcription process was performed at 37 °C for 5 h. After the transcription product was incubated with 50 nM MG for 30 min, the emission spectra were obtained.

### 2.4. Selectivity Evaluation of the Assay

For the selectivity evaluation of the assay, 1 mg/mL of reference proteins (including BSA and CRP) or 20 U/mL reference enzymes (such as Bst2.0 WarmStart, TdT, and Nt. AlwI) were added into the PNK buffer respectively instead of 1 U/mL T4 PNK. The concentration of other proteins/enzymes was much higher than that of T4 PNK. The ligation and the transcription processes were the same as in the typical experiment demonstrated above.

### 2.5. Kinase Inhibitor Screening

For T4 PNK inhibitor screening, we chose Na_2_HPO_4_ and (NH_4_)_2_SO_4_ as the model inhibitors. To confirm that the inhibitors did not affect the other two enzymes, we utilized a 5′ phosphorylated promoter probe in the control assays. T4 PNK was incubated with 100 mM Na_2_HPO_4_ and 50 mM (NH_4_)_2_SO_4_, respectively, before the 5′ phosphorylated promoter and ATP were added to the control assays.

The procedure of the T4 PNK inhibition experiment was similar to that of T4 PNK activity detection except for the addition of different concentrations of inhibitors together with 0.5 U/mL T4 PNK.

### 2.6. Determination of T4 PNK Activity in Diluted Cell Lysates

Hepatocellular carcinoma LM3 (HCC LM3) cells were purchased from the Cell Bank of Type Culture Collection of the Chinese Academy of Sciences (Shanghai, China). Cells were cultured in a DMEM medium supplemented with 10% FBS, penicillin (100 U/mL), and streptomycin (100 μg/mL) at 37 °C in a humidified atmosphere containing 5% CO_2_. A total of 2 × 10^7^ LM3 cells were washed 3 times with a PBS buffer before being suspended in 20 mL of an ice-cold buffer (70 mM Tris-HCl, 10 mM MgCl_2_, 50% glycerol, PH 8.0). Then the cells underwent ultrasound in the conditions of 2 s ultrasonic time, 5 s interval time, and 10 min total working time and were then stored at −20 °C before usage. The determination of T4 PNK activity in the diluted cell lysates experiment was similar to that of T4 PNK activity detection except for mixing 2 μL cell lysates with T4 PNK.

## 3. Results and Discussion

### 3.1. Sensing Principle of the Proposed Assay for T4 PNK Activity

**Scheme 1:**
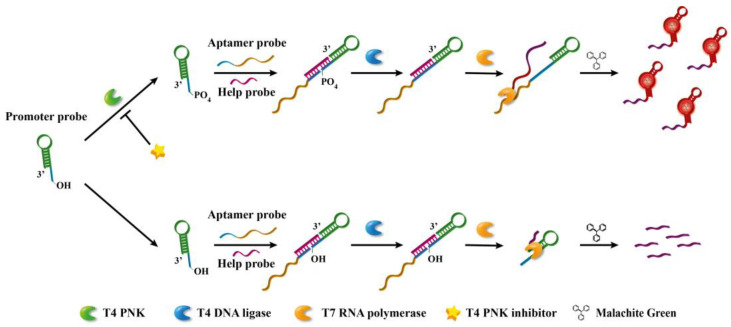
Schematic illustration of T4 PNK activity detection and inhibitor screening by combining a designed ligation-triggered T7 transcriptional amplification system and the light-up malachite green aptamer.

The principle of this T4 PNK activity detection strategy based on ligation-triggered transcription is outlined in Scheme 1. Our strategy involved three probes: the promoter probe, the aptamer probe, and the help probe. The promoter probe is composed of a stem-loop-shaped T7 promoter sequence and a hanging 5′ end region whose sequence is complementary to the 5′ ends of the help probe. Similar to the promoter probe, the 3′ end region of the aptamer probe is complementary to the 3′ ends of the help probe, with the rest sequence (so-called MGA template) complementary to the MGA. In addition, the promoter probe contains a 5′-hydroxyl group which can be phosphorylated by T4 PNK. The 5′ phosphorylated promoter probe could form a ligatable nicking site together with the aptamer probe and the help probe. After ligation by the T4 DNA ligase, we obtained intact transcribable template sequences containing a T7 promoter and the MGA template, whose amount was positively correlated with T4 PNK activity. Subsequently, T7 RNA polymerase continuously catalyzed the synthesis of MGAs that specifically bound to MG from the above intact template sequences. As a result, the mounting fluorescence intensity suggested an increasing T4 PNK activity. Thus, we could evaluate the relative activity of PNK by measuring the fluorescence intensity. On the other hand, if the phosphorylation procedure of the promoter probe’s 5′-hydroxyl group was inhibited, the amount of ligatable nicking sites and complete transcribable templates was reduced, resulting in a decreasing number of MGAs and weakened fluorescence intensity. Thus, this proposed assay for T4 PNK activity could also be employed for the T4 PNK inhibitor screening.

### 3.2. Feasibility Verification of the Strategy

**Figure 1:**
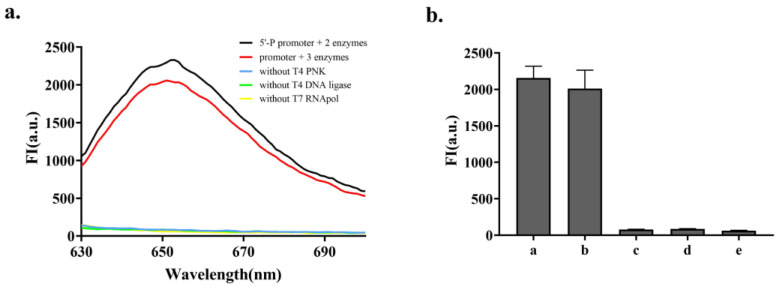
Fluorescence spectrum of the sensing system containing 200 nM promoter probes, aptamer probes, and help probes in the presence of different concentrations of T4 PNK (blue: 0 and red: 5 U/mL), 20 U/mL T4 DNA ligase, and 100 U/mL T7 RNA polymerase. The black, green, and yellow curves are for the control experiment using 200 nM purchased phosphorylated promoter probes instead of unphosphorylated promoter probes in the absence of T4 PNK, T4 DNA ligase, T7 RNA polymerase. Histograms a, b, c, d, e in (**b**) correspond to black, red, blue, green and yellow curves in (**a**) respectively. The error bars represent the standard deviation (SD) across three repetitive experiments over the blank response.

To verify the feasibility of this T4 PNK activity detection strategy based on a ligation-triggered transcription, a 5′-phosphorylated promoter probe was synthesized as the positive control. As shown in Figure 1, with the existence of the T4 DNA ligase and T7 RNA polymerase but not T4 PNK, the 5′-phosphorylated promoter probes (the black curve in Figure 1a and histogram a in Figure 1b) showed an extremely enhanced fluorescence compared to the same amount of unphosphorylated promoter probes (the blue curve in Figure 1a and histogram c in Figure 1b). However, when the unphosphorylated promoter probes reacted with 5 U/mL T4 PNK for 30 min before the ligation process, we observed a fluorescence intensity similar to that of the 5′-phosphorylated promoter probes (Figure 1a red curve and Figure 1b histogram b). This is owed to the dependence of the T4 DNA ligase on 5′-phosphate in duplex DNA when catalyzing the formation of a phosphodiester bond, while the function of T4 PNK is to phosphorylate 5′ OH of the promoter probe. At present, there are conflicting reports on the ability of T7 RNA polymerase to bypass the nicking site in the template strand of DNA [32], so we next verified the necessity of the ligation process for the generation of MGAs. When tested under the same condition except for the existence of T4 DNA ligase, the fluorescence intensity was bitterly lower without T4 DNA ligase (Figure 1a red curve and Figure 1b histogram b) compared with that of the T4 DNA ligase (Figure 1a green curve and Figure 1b histogram d). The above results illustrate that two separate probes could not be used as templates for transcription. Moreover, T7 RNA polymerase could not bypass the nicking site of the template regardless of the presence of 5′ phosphate. Similarly, when T7 RNA polymerase was not present, there was almost no enhancement in the fluorescence intensity at the emission wavelength of MGA (Figure 1a yellow curve and Figure 1b histogram e), indicating that T7 RNA polymerase is also indispensable for this strategy. Thus, with the assistance of the T4 DNA ligase and T7 RNA polymerase, MGA was only generated in the presence of T4 PNK, whose amount positively correlated with the 5′ phosphate catalyzed by T4 PNK.

### 3.3. Optimization of Experimental Conditions

**Figure 2:**
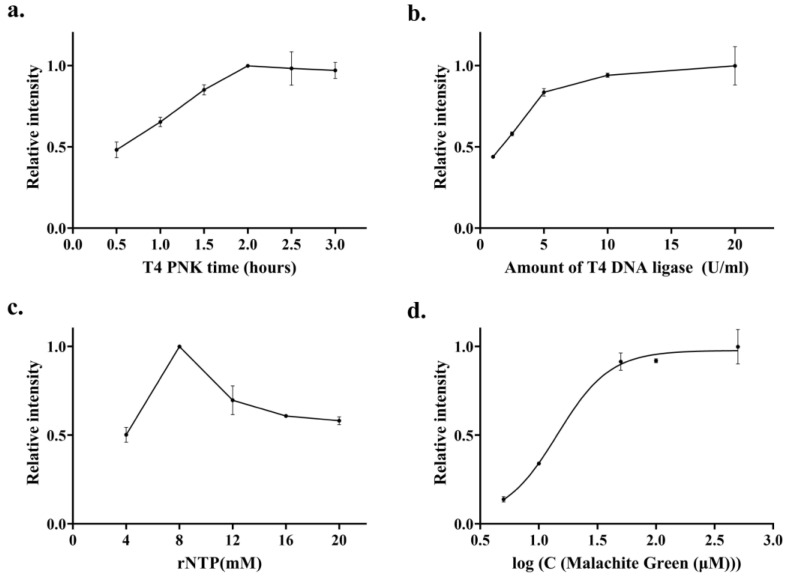
(**a**) Optimization of phosphorylation time. The concentrations of T4 PNK, T4 DNA ligase, and T7 RNA polymerase were 2 U/mL, 20 U/mL, and 100 U/mL, respectively. The concentrations of rNTP and malachite green were 4 mM and 100 μM. (**b**) Optimization of T4 DNA ligase concentration. The concentrations of T4 PNK and T7 RNA polymerase were 2 U/mL and 100 U/mL, respectively. The concentrations of rNTP and malachite green were 4 mM and 100 μM. (**c**) Optimization of rNTP concentration. The concentrations of T4 PNK, T4 DNA ligase, and T7 RNA polymerase were 2 U/mL, 10 U/mL, and 100 U/mL, respectively. The concentrations of malachite green were 100 μM. (**d**) Optimization of malachite green concentration. The concentrations of T4 PNK, T4 DNA ligase, and T7 RNA polymerase were 2 U/mL, 10 U/mL, and 100 U/mL, respectively. The concentrations of rNTP and malachite green were 8 mM. The fluorescence intensities at 650 nm were all normalized to 1. The error bars represent the standard deviation (SD) across three repetitive experiments over the blank response.

In order to obtain better detection efficiency, we optimized the experimental conditions, including the T4 PNK reaction time, the amount of T4 DNA ligase, the concentration of rNTP, and the concentration of MG. The results are displayed in the form of relative fluorescence intensity, which is the difference between the final fluorescence intensity and the background fluorescence intensity. It is shown in Figure 2a that, with the extension of the T4 PNK reaction time, relative fluorescence intensity gradually increased, reaching a plateau at 2 h. Therefore, we incubated T4 PNK and the promoter probes for 2 h in subsequent experiments. To obtain a wider linear detection range, it was necessary to ensure that all phosphorylated probes were connected with the aptamer probes, so we optimized the concentration of the T4 DNA ligase introduced into the system. As shown in Figure 2b, the relative intensity increased following the successive addition of T4 DNA ligase until the amount of T4 DNA ligase reached 10 U/mL. As a consequence, 10 U/mL was chosen for the following experiment. Next, we determined the most appropriate rNTP concentration, and the result is shown in Figure 2c. With the increase in rNTP concentration, the relative intensity first increased and then decreased, reaching the highest at an rNTP concentration of 8 mM. This phenomenon that the transcription product first increased and then decreased with the increase in rNTP concentration is consistent with previous reports [33,34]. Finally, we determined 8 mM as the rNTP concentration that would ensure the highest transcription efficiency. The results in Figure 2d indicate that the gradual addition of MG led to a gradual increase in the relative intensity. After the generated aptamers were saturated by MG molecular, the plateau was reached when the MG concentration reached 50 μM. Thus, the optimal MG concentration was set to 50 μM.

### 3.4. Quantitative Detection of T4 PNK Activity

**Figure 3:**
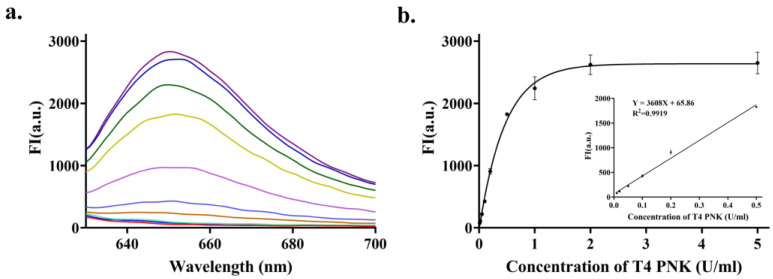
(**a**) Fluorescence emission spectrum of the assay upon addition of different units of T4 PNK (from bottom to top): 0, 0.01, 0.02, 0.05, 0.1, 0.2, 0.5, 1, 2, 5 U/mL. (**b**) Calibration curve for T4 PNK detection. The error bars represent the standard deviation (SD) across three repetitive experiments over the blank response.

In order to evaluate the limit of detection (LOD), a series of samples containing different concentrations of T4 PNK (ranging from 0.01 U/mL to 5 U/mL) were investigated under the optimized condition. The fluorescence spectrum for varying concentrations of the target was recorded and is depicted in Figure 3a. As expected, the fluorescence intensity gradually increased with the increasing T4 PNK concentration. There was a good linear range between 0 and 0.50 U/mL, and then the fluorescence intensity reached a plateau at 2.00 U/mL. The calibration curve is plotted in Figure 3b, whose corresponding linear equation was Y = 3608x + 65.86 with an R square of 0.9919. Using the calculation formula LOD = 3 σ/m (σ represents the standard deviation of the blank sample, and m represents the slope of the standard curve), the limit of detection for PNK was evaluated at 0.01 U/mL, indicating that our method has promising clinical application. The analytical performance of our T7 transcription and MGA combined methodology was compared with other fluorescent assays previously reported, and the results are summarized in Table 2. Due to the robustness of T7 transcriptional amplification and the brightness of the light-up MGA system, our label-free assay for PNK achieved a satisfactory sensitivity, which was better than or equal to all the listed methods. In addition, the isothermal and homogeneous reaction properties of our method obviated the necessity for a precise and expensive thermal cycler and rendered it facile to realize and thereby enhance its applicability in less developed regions.

### 3.5. Selectivity Evaluation of the Assay

**Figure 4:**
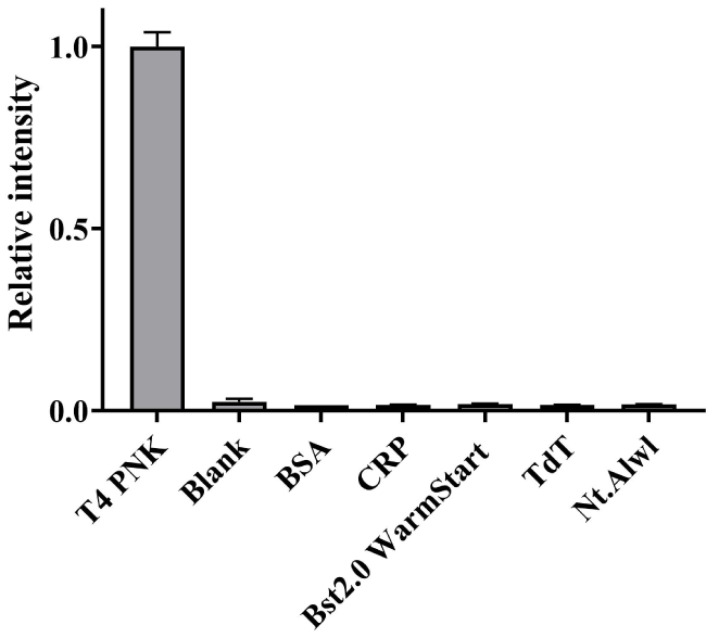
The selectivity of the label-free detection of T4 PNK activity based on the ligation-triggered transcription. The fluorescence intensities at 650 nm were all normalized to 1. The error bars represent the standard deviation (SD) across three repetitive experiments over the blank response.

Achieving satisfactory specificity is an essential requirement for an assay to be deemed suitable for application in complex clinical samples. In order to evaluate the specificity of our methodology, common proteins, such as BSA and CRP, as well as widely utilized tool enzymes, including Bst2.0 WarmStart DNA polymerase, Terminal deoxynucleotidyl transferase (TdT), and Nt. AlwI endonuclease was recruited to challenge the assay with the optimized condition. To ensure the reliability of the specificity verification, we utilized relatively high concentrations of reference proteins (1 mg/mL) or enzymes (20 U/mL) in this study. As shown in Figure 4, except for the significantly enhanced fluorescence intensity of the T4 PNK group, the fluorescence intensity of groups with other proteins or enzymes was almost unanimous to that of the blank control. The above results indicate that our assay had satisfactory specificity so that it could be used for the detection of biological samples.

### 3.6. Inhibition Evaluation of T4 PNK Activity

PNK is not only an important biomarker but also a key target for tumor treatment, for the inhibition of PNK may disturb the phosphorylation-dependent rehabilitation of DNA damage and have a synergistic effect on tumor treatment methods based on destroying tumor DNA. Subsequently, we experimentally verified that our label-free detection of PNK activity via MGA could be used to screen PNK inhibitors. First, we chose two common inhibitors that were reported previously as model inhibitors [41,42,43]. The inhibition may be attributed to the effect of high salt concentrations on the conformation of T4 PNK [44]. Because the phosphorylated products were diluted after the phosphorylation process, the effects of various inhibitors on the activities of T4 DNA ligase and T7 RNA polymerase could be reduced. Nevertheless, we tested whether the diluted inhibitors we chose affected the other two enzymes in case the inhibitors did not only affect the activity of T4 PNK. As shown in Figure S1 (in Supplementary Material), there was only a slight difference in the fluorescence response between the control group and the two model inhibitor groups, which indicates that the influence of Na_2_HPO_4_ and (NH4)_2_SO_4_ on T4 DNA ligase and T7 RNA polymerase was negligible. Because inhibitors only affect the activity of T4 PNK, this strategy could also be used for the screening of inhibitors based on other principles except for the model inhibitors we chose.

**Figure 5:**
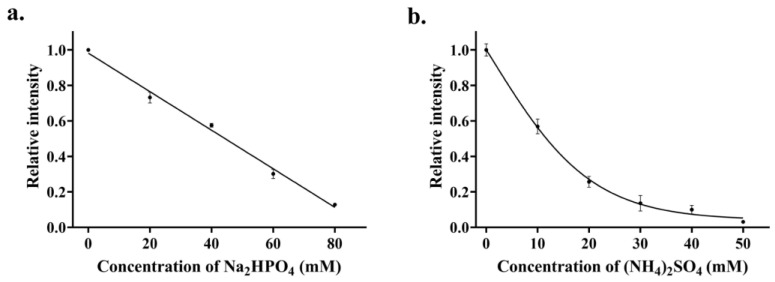
(**a**) Inhibition effects of Na_2_HPO_4_ on phosphorylation. (**b**) Inhibition effects of (NH_4_)_2_SO_4_ on phosphorylation. The fluorescence intensities at 650 nm were all normalized to 1. The error bars represent the standard deviation (SD) across three repetitive experiments over the blank response.

Next, we analyzed the effect that different concentrations of inhibitors had on T4 PNK activity. As can be seen in Figure 5a,b, the fluorescence intensity gradually decreased with the incessant addition of Na_2_HPO_4_ and (NH_4_)_2_SO_4_. The half-maximal inhibition value (IC50) was the concentration of the inhibitors inhibiting half of the maximum fluorescent intensity. The IC50 of Na_2_HPO_4_ and (NH_4_)_2_SO_4_ were approximately 44.15 mM and 11.44 mM, which is similar to that reported in the previous literature [36]. The above results showed that our method could be used to screen T4 PNK inhibitors reliably.

### 3.7. PNK Activity Detection in Diluted Cell Extracts

**Figure 6:**
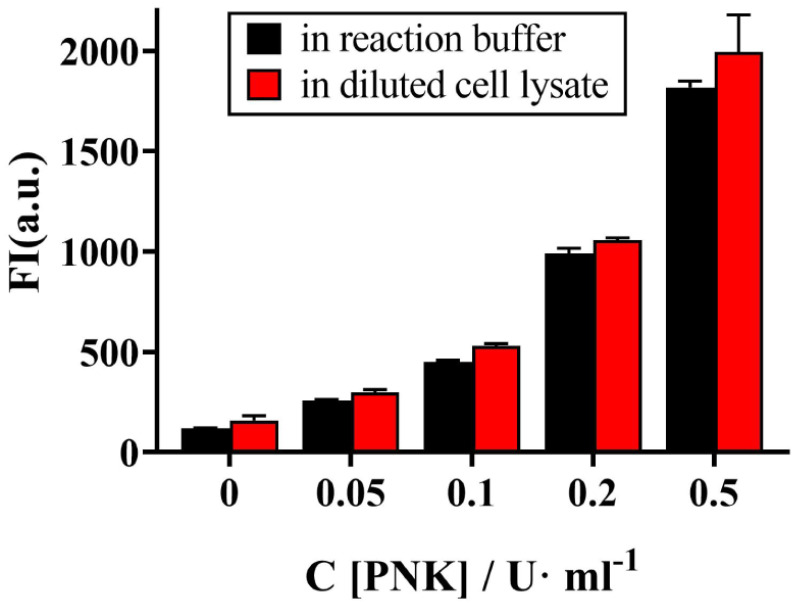
Fluorescence intensity for the label-free detection of T4 PNK activity based on the ligation-triggered transcription in reaction buffer and diluted cell lysates spiked with different concentrations of T4 PNK. The error bars represent the standard deviation (SD) across three repetitive experiments.

To evaluate the robustness of our strategy, we further analyzed its practical applicability in HCC LM3 cell lysates, which were employed to simulate the complex biological matrix. The fluorescence intensity increased with the increase in T4 PNK concentration in the diluted cell lysate (Figure S2a). The fluorescent intensity exhibited a linear correlation with the PNK concentration from 0.05 to 0.5 U/mL (Figure S2b). Moreover, as shown in Figure 6, the detection results in diluted cell lysates were consistent with those in the pure reaction buffer. Thus, our T4 PNK activity detection assay based on a ligation-triggered transcription has good reliability in a complex biological matrix represented by cell lysates, which means it has great potential for clinical application.

## 4. Conclusions

In summary, we herein developed a simple fluorescence sensing system for T4 PNK activity detection and inhibitor screening by combining a designed ligation-triggered T7 transcription system and a light-up MGA. This system shares several distinct advantages. First of all, we made use of the excellent sensitivity of MGA as a sensing element; we achieved a simple, economic, label-free assay for T4 PNK with a LOD of 0.01 U/mL. Secondly, this isothermal amplification-based assay has no need of large and expensive thermal cyclers, which could enable the application of this method in resource-limited settings and for point-of-care analysis. In addition, this method takes advantage of the robust signal amplification ability of the T7 transcriptional system for signal amplification, reducing the non-specific amplification caused by high primer concentration. The ability to screen T4 PNK inhibitors in this method was also successfully evaluated, with Na_2_HPO_4_ and (NH4)_2_SO_4_ as the model inhibitors. Furthermore, this was successfully applied in complex biological samples such as diluted cell lysates, implying its great potential in the biomedical field.

## Figures and Tables

**Table 1:** Sequences of the oligonucleotides used in this work.

Name	Sequence (5′–3′)
Promoter probe	AGGTTACAGATGTGGCCCTATAGTGAGTCGTATTAATTTCGCGACAACACGCGAAATTAATACGACTCACTATAGGG
5′ P-promoter probe	P-AGGTTACAGATGTGGCCCTATAGTGAGTCGTATTAATTTCGCGACAACACGCGAAATTAATACGACTCACTATAGGG
Aptamer probe	GGATCCATTCGTTACCTGGCTCTCGCCAGTCGGGATCCTGATAGCTTGACAAC
Help probe	CCACATCTGTAACCTGTTGTCAAGCTATCA

**Table 2:** Comparison of the analytical performances of different detection methods for T4 PNK activity.

Sensing Strategy	Detection Limit	Ref.
G-quadruplex-based luminescent assay coupled with λ exo cleavage reaction	0.05 U/mL	[35]
Graphene oxide-based fluorescent assay assisted by λ exo	0.05 U/mL	[36]
Fluorescent assay with dsDNA-templated CuNPs as the fluorescence indicator	0.49 U/mL	[37]
Colorimetric assay based on horseradish peroxidase-mimicking DNAzyme combined with λ exonuclease cleavage	0.06 U/mL	[12]
Electrochemical assay by TiO2 nanotube array as a peroxidase mimic	0.15 U/mL	[38]
Electrochemical Assay Based on Dual-Signaling Amplification Coupled with Exonuclease Reaction	0.02 U/mL	[15]
Ferrocene-functionalized SWCNT for electrochemical detection	0.01 U/mL	[14]
Fluorescent assay by a Singly Labeled DNA-Hairpin Smart Probe Coupled with λ Exonuclease Cleavage	0.04 U/mL	[39]
Fluorescent assay based on β-cyclodextrin polymer combined with an exonuclease reaction	0.02 U/mL	[40]
Fluorescent detection assay via malachite green aptamer generated from ligation-triggered transcription	0.01 U/mL	This work

## Data Availability

The completed data of this study are available from the corresponding author.

## Supplementary Materials

The following supporting information can be downloaded at: https://www.mdpi.com/article/10.3390/bios13040449/s1, Figure S1: Inhibition effects of Na_2_HPO_4_ and (NH_4_)_2_SO_4_ on T4 DNA ligase and T7 RNA polymerase. Figure S2: Fluorescence emission spectrogram and calibration curve plot for T4 PNK detection in diluted cell lysate.
