# Supplementary material for: Label-Free Detection of T4 Polynucleotide Kinase Activity and Inhibition via Malachite Green Aptamer Generated from Ligation-Triggered Transcription
Source: Biosensors (Basel). 2023 Mar 31;13(4):449. doi: 10.3390/bios13040449 (PMC10135927; doi:10.3390/bios13040449)
Supplement: Supplementary file 1 [file biosensors-13-00449-s001.zip › biosensors-2167392-supplementary.pdf]

biosensors

MDPI

Supplementary Material

Label-Free Detection of T4 Polynucleotide Kinase Activity and Inhibition via Malachite Green Aptamer Generated from Ligation-Triggered Transcription

Jingyi Si [1], Wei Zhou [2], Ying Fang [1], Da Zhou [1], Yifan Gao [1], Qunyan Yao [1], Xizhong Shen [1] and Changfeng Zhu [1,*]

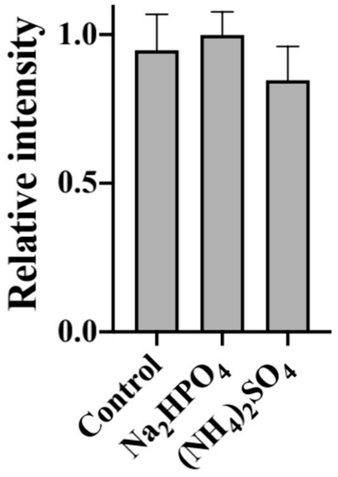

Figure S1. Inhibition effects of Na_2HPO_4 and $(NH_4)_2SO_4$ on T4 DNA ligase and T7 RNA polymerase.

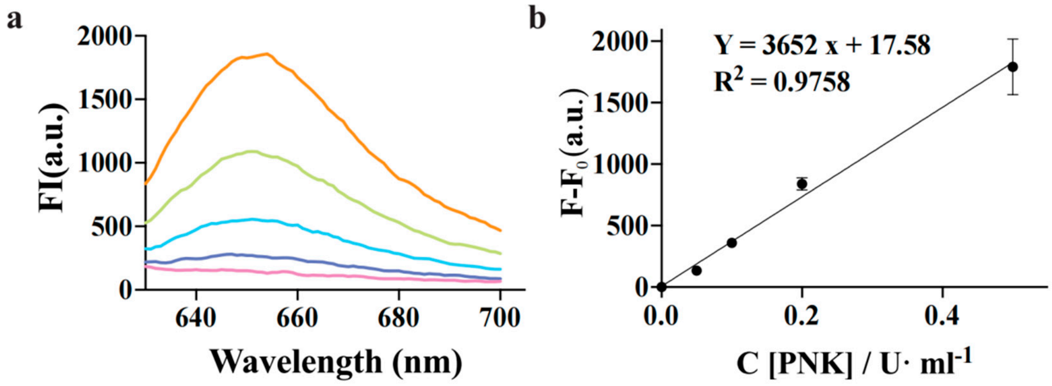

Figure S2. (**a**) Fluorescence emission spectrum of the assay upon addition of different units of T4 PNK in diluted cell lysate (from bottom to top): 0, 0.05, 0.1, 0.2, 0.5 U/mL. (**b**) Calibration curve for T4 PNK detection in diluted cell lysate. The error bars represent the standard deviation (SD) across three repetitive experiments over the blank response.
